# Human resources needs for universal access to antiretroviral therapy in South Africa: a time and motion study

**DOI:** 10.1186/1478-4491-10-39

**Published:** 2012-10-30

**Authors:** Jan AC Hontelez, Marie-Louise Newell, Ruth M Bland, Kristen Munnelly, Richard J Lessells, Till Bärnighausen

**Affiliations:** 1 Africa Centre for Health and Population Studies, University of KwaZulu-Natal, Mtubatuba, South Africa; 2 Department of Public Health, Erasmus MC, University Medical Centre Rotterdam, Rotterdam, Netherlands; 3 Department of Primary and Community Care, Radboud University Nijmegen Medical Center, Radboud, Netherlands; 4 College of Health Sciences, Medical Faculty, University of Glasgow, Glasgow, UK; 5 Department of Clinical Research, London School of Hygiene and Tropical Medicine, London, UK; 6 Department of Global Health and Population, Harvard School of Public Health, Boston, USA

**Keywords:** Human resources for health, HIV, South Africa, Antiretroviral treatment

## Abstract

**Background:**

Although access to life-saving treatment for patients infected with HIV in South Africa has improved substantially since 2004, treating all eligible patients (universal access) remains elusive. As the prices of antiretroviral drugs have dropped over the past years, availability of human resources may now be the most important barrier to achieving universal access to HIV treatment in Africa. We quantify the number of HIV health workers (HHWs) required to be added to the current HIV workforce to achieve universal access to HIV treatment in South Africa, under different eligibility criteria.

**Methods:**

We performed a time and motion study in three HIV clinics in a rural, primary care-based HIV treatment program in KwaZulu-Natal, South Africa, to estimate the average time per patient visit for doctors, nurses, and counselors. We estimated the additional number of HHWs needed to achieve universal access to HIV treatment within one year.

**Results:**

For universal access to HIV treatment for all patients with a CD4 cell count of ≤350 cells/μl, an additional 2,200 nurses, 3,800 counselors, and 300 doctors would be required, at additional annual salary cost of 929 million South African rand (ZAR), equivalent to US$ 141 million. For universal treatment (‘treatment as prevention’), an additional 6,000 nurses, 11,000 counselors, and 800 doctors would be required, at an additional annual salary cost of ZAR 2.6 billion (US$ 400 million).

**Conclusions:**

Universal access to HIV treatment for patients with a CD4 cell count of ≤350 cells/μl in South Africa may be affordable, but the number of HHWs available for HIV treatment will need to be substantially increased. Treatment as prevention strategies will require considerable additional financial and human resources commitments.

## Introduction

With about 22.5 million people living with HIV [1], the disease remains one of the most important health problems in sub-Saharan Africa (SSA). Antiretroviral therapy (ART) significantly improves the survival and quality of life of people infected with HIV [2-4]. In June 2011, the United Nations General Assembly High Level Meeting on AIDS adopted a political declaration of achieving ‘universal access’ to HIV treatment by 2015 [5]. South Africa has the largest population infected with HIV worldwide (nearly 6 million people) [1], but access to HIV treatment remains far from universal; under the 2010 WHO ART eligibility criteria [6], ART coverage was estimated to be 55% in South Africa in 2010 [7]. Because the cost of antiretroviral medicines has dropped dramatically over the past decade, the availability of well-trained health workers may now be the most important barrier to providing life-saving HIV treatment to those in need [8-10]. Based on a review of scientific publications and government documents, George *et al.* concluded that in 2007/2008 there was a total shortage of 79,791 health workers in South Africa’s public sector [11].

The current WHO guidelines recommend that ART should be initiated when CD4 cell counts drops to ≤350 cells/μl [6] to improve the health of both the individual taking ART (by decreasing mortality and morbidity) and of the community members uninfected with HIV (by reducing onward transmission of the virus) [12]. South Africa changed its treatment eligibility criteria for adults in 2010 to include those with CD4 cell counts of 200 to 350 cells/μl if they were co-infected with tuberculosis or pregnant [13], and further relaxed eligibility criteria in August 2011 to ART for all HIV-infected people with CD4 cell counts of ≤350 cells/μl [14]. More recently, it has been argued that treatment should be given to all HIV-infected people, regardless of their CD4 cell count, as a ‘treatment as prevention’ strategy to substantially reduce HIV transmission [15,16]. The National Strategic Plan on HIV, STIs and TB for the period 2012 to 2016 states that all HIV treatment in South Africa should be delivered through decentralized, nurse-led primary health care (PHC) HIV clinics [17]. Treatment initiation is performed by doctors who rotate between clinics, while professional nurses and HIV counselors perform the follow-up visits. Recently, the South African government changed its guidelines to also allow nurse-initiated ART [18]. Health workers are usually employed by the Department of Health on a contract basis, and payment of salaries is based on full-time equivalents (FTEs) on a monthly basis.

We used novel data on ART task times obtained in a time and motion study to estimate the number of additional HIV health workers (HHWs) required to achieve universal access to ART in South Africa. We determined the effects of alternative ART delivery models on the additional number of HHWs required for universal ART access, and the financial resources needed to pay the salaries of those HHWs.

## Methods

### Ethics approval

We obtained consent for this study from the local Department of Health. This study received ethics approval from the Biomedical Research Ethics Committee of the University of KwaZulu-Natal (ethics certificate number BF109/09). Written consent was obtained from all HHWs observed.

### Data collection

We performed a time and motion study (a direct and continuous observation of tasks, using a timekeeping device to record the time taken to accomplish a task [19]) in the Hlabisa HIV Treatment and Care Programme in KwaZulu-Natal, South Africa, which is a partnership between the local Department of Health and a Wellcome Trust-funded research center based in the community (the Africa Centre for Health and Population Studies, University of KwaZulu-Natal) [20]. HIV treatment is delivered within the program through 17 PHC HIV clinics and one district hospital. The professional nurses and trained HIV counselors perform tasks exclusively related to HIV treatment and care. Doctors visit clinics on a scheduled rotation (usually one weekly visit per clinic) to start new patients on ART and to review cases of treatment failure, drug toxicity, and other complications. The Hlabisa sub-district has a total population of 228,000 and an adult HIV prevalence of 28% [21]. ART uptake in the area is high, and by June 2011, nearly 17,000 people had been initiated on ART [22].

We randomly selected three PHC HIV clinics (clinics A to C) within the treatment program for observation. A single observer was then randomly assigned to a different HHW (in one of the three categories: doctor, nurse, or treatment counselor) on different, randomly assigned calendar days within the observation period. No HHW was observed more than once. Activities were timed and recorded for each separate patient contact, and subsequently entered into a spreadsheet (Excel; Microsoft Corp., Redmond, WA, USA). Data were collected by a single observer trained in quantitative and qualitative data collection, and the observer was closely supervised by three doctors and two professional nurses. The observer was given written instructions on how to keep and record time for different types of tasks, and was trained in two stages. The initial stage involved observing the work days of different HHWs without recording any tasks, in order to allow the observer to become familiar with the work routine. Next, a pilot study was conducted in which tasks were observed and recorded, and subsequently coded. The pilot data were checked for errors or inconsistencies, and the study protocol was improved, based on the pilot findings. During the final data collection, data were continuously entered and checked by the supervising doctors.

Two investigators independently coded the recorded activities into pre-defined categories: 1) direct patient contact (talking to patient; writing; writing and talking; venepuncture; physical examination; dispensing medication); 2) indirect patient contact (discussing clinical or work-related issues with other HHWs; performing work-related paperwork or administration; contacting health workers in other healthcare facilities, such as hospitals, for patient referral); and 3) other (breaks; idle time; unaccounted time). Categories (1) and (2) are times allocated to perform tasks within the job description of the particular HHW, while category (3) contains breaks and idle time. It is important to note that ‘breaks’ and ‘idle time’ do not necessarily imply wasted or unproductive time. Breaks may serve an important purpose in allowing the HHW to maintain productivity when performing tasks in categories (1) and (2). The final assignment of category codes was determined in discussion between the two investigators and, when conflicting assignments could not be resolved, through discussion with a third investigator.

### Data analysis

We calculated time per patient, duration of a work day, and the proportion of the work day spent on direct and indirect patient contact, and other activities. We used one-way ANOVA to test differences in average time per patient and average duration of work days between HHWs and clinics. All analyses were performed using SAS software (version 9.0; SAS Institute Inc., Cary, NY, USA).

### Human resources needs and salary costs of scaling up

Next, we estimated the additional number of HHWs and the salary costs required for scaling up ART from the current coverage level to universal access within a year, and maintaining these new patients on ART for a period of 12 months after initiation, using the following eligibility criteria: 1) ART at CD4 cell counts of ≤200 cells/μl; 2) ART at CD4 cell counts of ≤350 cells/μl for patients co-infected with tuberculosis or pregnant; 3) ART at CD4 cell counts of ≤350 cells/μl for all; 4) ART at CD4 cell counts of ≤500 cells/μl; and 5) ART for all HIV-infected people. In addition, we considered the impact of the additional salary costs for these HHWs on the total HIV sector budget for South Africa. We used estimates from the most recent *UNAIDS Report on the Global HIV Epidemic 2010* and the *WHO, UNAIDS and UNICEF Progress Report 2010* on universal access to obtain the current number of people living with HIV, receiving ART, and eligible for ART in South Africa under different treatment thresholds [1,23].

At the time of this study, South African guidelines stated that every treatment initiation visit should include a contact with a doctor, nurse, and a counselor, and that all ART initiations should be conducted by doctors. Since then, the treatment guidelines in South Africa have changed, allowing ART initiation by nurses trained in ART delivery. A patient on ART should return for a routine clinic visit with a nurse and a counselor every month. When treatment stabilizes, the re-visit frequency can be reduced to once every 2 months [24]. For the purposes of this study, we assumed that all recorded doctor time represented initiation visits, and all recorded nurse and counselor time represented both the time spent during routine clinic visits and treatment initiation visits. A full-time HHW is assumed to have 20 work days per month (based on a total of 22 work days per month, after accounting for holidays and sick leave), and the average number of hours in a work day spent on patient contact (direct and indirect) was derived from the time and motion study results. We used the average salary of a given HHW in the local HIV treatment program as of 2010: 38,733 South African rand (ZAR) (or US$ 5,869, assuming an exchange rate of ZAR 6.6 to US$ 1.0 [25]) per month for doctors; ZAR 20,013 (US$ 3032 per month for nurses; and ZAR 6,024 (US$ 913) per month for counselors. The total South African HIV sector budget was US$ 2.1 billion in 2009 [1] (or ZAR 14 billion).

In the baseline scenario, we estimated the total number of doctor-months required for universal coverage by: 1) measuring the time taken for a single treatment initiation; 2) multiplying this by the number of required initiations; and 3) dividing this by the number of work hours in a month. We then translated the total number of doctor-months into the number of FTEs on an annual basis that would be needed for initiating all eligible patients in one year, and the number of doctors per 1,000 initiations. In an alternative scenario, we assumed that all initiations were conducted by nurses.

In the baseline scenario, we further assumed that all new patients return on a monthly basis for our study period (one year) for a routine clinic visit with a nurse and a counselor. We estimated the total number of nurse-months and counselor-months needed to initiate and maintain all eligible patients on treatment for one year by: 1) measuring the time taken for one visit; 2) multiplying this time with the number of newly initiated patients required for universal access; and 3) dividing this value by the number of work hours in a month. We then translated the total number of nurse-months and counselor-months into the number of FTEs needed for universal ART coverage.

### Sensitivity and scenario analysis

For our baseline estimate we assumed constant returns to scale. However, the efficiency of ART delivery may be a function of scale [26]; that is, efficiency may increase (economies of scale) or decrease (diseconomies of scale) as the total number of patients increases. Although the empirical evidence on the shape of scale function in HIV treatment is limited [27,28], Kumaranayake *et al.* reported in a review of the evidence that if the number of patients increases by a factor of 23, the costs per ART for each patient will be reduced by 27% to 73% [26]. However, it is also plausible that returns to scale could be decreasing; that is, we would find increasing costs per patient as the number of patients grows. For instance, within the national South African HIV treatment program the clinics that would need to be added to the ART delivery infrastructure to reach remote and rural populations might operate below their full capacity because of limited ART patient load, owing to the low population density in remote and rural areas. In fact, the clinics in which ART was initially rolled out were more likely to have been located in more densely populated, urban areas, and thus are more likely to operate at capacity. Thus, as the program is scaled up, the fixed costs per patient may increase, implying diseconomies of scale. The running costs per patient may also increase with scale, as the health-system efforts required to motivate patients to begin and continue taking ART may be lower for patients who accessed the ART program at earlier stages of the scale-up than for those who accessed the program at later stages. We thus assessed two scenarios to measure the scale effects: one assuming increasing returns to scale, the other assuming decreasing returns to scale. In both cases, we assumed that the scale effects followed an exponential distribution. The equations were:

patients per HHW = EXP(0.03858 × F)

and

patients per HHW = EXP(−0.03858 × F),

for economies and diseconomies of scale, respectively, where *F* = a constant number of patients per HHW across scale. In addition, we examined the sensitivity of our results to changes in the average ‘time per patient’ and ‘duration of work day’ by using the times and durations of either the least efficient or the most efficient clinic in our estimations.

Finally, we performed a scenario analysis on the effect of alternative models of delivering ART on the HHW:patient ratio and overall salary costs. Nurse-initiated treatment has been allowed in South Africa in recent years [24]; therefore, we performed an additional analysis in which nurses were assumed to perform all initiations, at the same productivity level as doctors [18]. In addition, we assumed the following alternative delivery models: i) decreased frequency of routine clinic visits (once every 2, 3, or 4 months); and ii) decreased frequency of nurse-attended routine clinic visits (once every 2, 3, or 4 months).

## Results

Table 1 gives an overview of the baseline characteristics of the time and motion data. In total, thirteen HHWs (six nurses, four counselors, and three doctors) were observed over the period 12 August to 1 September 2009. The total number of patient visits observed was 334. An average work day lasted 6.3 hours, and the average duration of work days differed significantly between the busiest and least rural clinic (clinic B) and the least busy and most rural clinic (clinic C) in our sample (7.3 versus 4.9 hours; *P* = 0.01). The observed HHW with the shortest observed work-day duration was a counselor in clinic C, who spent only 20% of the work day on direct patient contact; this counselor used 44% of the work day to perform administrative work and 36% on breaks and idle time. This time distribution was probably due to the fact that the patient load in this rural clinic was relatively low. There were no significant differences in the time per patient between the three clinics (*P* = 0.47) or between the different HHWs (*P* = 0.24). On average, a nurse-visit took 10 minutes (95% CI 6 to 13 minutes), a counselor-visit 14 minutes (95% CI 9 to 19 minutes), and a doctor-visit 13 minutes (95% CI 9 to 16 minutes).

**Table 1:** Baseline characteristics of the time and motion data

	**Overall**	**By clinic**		
		**Clinic A**	**Clinic B**	**Clinic C**
Observation days, n	13	5	4	4
Observation period	12 August to 1 September	19 to 26 August	12 to 18 August	27 August to 1 September
**HHWs observed, n**				
Nurse	6	2	2	2
Counselor	4	2	1	1
Doctor	3	1	1	1
Total patients observed, n	334	115	120	99
**Average duration of work day, hours**				
Overall	6.3	5.2	7.3	4.9
Nurse	7.1	5.2	7.3	5.3
Counselor	5.8	5.3	6.7	5.8
Doctor	5.4	5.1	7.7	3.4
**Proportion of time (%) spent on**				
*Direct patient contact*^a^				
Overall	83	86	87	76
Nurse	83	84	83	82
Counselor	74	87	82	20
Doctor	92	91	94	89
Indirect patient contact^b^				
Overall	9	7	9	10
Nurse	9	8	13	6
Counselor	13	6	8	44
Doctor	4	8	4	3
*Other*^c^				
Overall	9	7	5	14
Nurse	9	9	3	12
Counselor	13	7	10	36
Doctor	4	2	2	8
*Average time per patient, minutes (95% CI)*				
Overall	12 (9 to 14)	14 (9 to 18)	11 (8 to 13)	10 (5 to 16)
Nurse	10 (6 to 13)	12 (6 to 18)	12 (7 to 17)	7 (1 to 12)
Counselor	14 (9 to 19)	15 (7 to 23)	8 (5 to 11)	43 (2 to 83)
Doctor	13 (9 to 16)	15 (2 to 27)	15 (11 to 18)	9 (7 to 11)

HHW, HIV health worker.

^**a**^Direct patient contact included activities such as talking to patients, venepuncture, physical examination, and prescribing medication.

^b^Indirect patient contact included activities such as consultations, meetings with colleagues, and administrative work.

^c^’Other’ consisted of breaks and idle time between patients.

Estimates of ART coverage under different eligibility criteria are shown in Table 2. As of 2009, nearly 1 million people were on HIV treatment in South Africa [1]. Universal access to ART for HIV-infected people with CD4 cell counts of ≤350 cells/μl would require an additional 1.6 million initiations (a total of 2.6 million people on treatment), whereas for universal access to ART at CD4 cell counts of ≤500 cells/μl, 3.1 million additional initiations would be needed (a total of 4.1 million people on treatment). ART for all HIV-infected people would require 4.6 million additional initiations to achieve universal access (a total of 5.6 million people on treatment).

**Table 2:** **Coverage indicators South Africa**^a^

	**Coverage indicators in South Africa**		
	**Point estimate**	**Low estimate**	**High estimate**
Receiving ART, n	971,566	NA	NA
Needing ART, n			
CD4 ≤200	1,700,000	1,500,000	2,000,000
CD4 ≤350 (TB or pregnant); CD4 ≤200 (all other HIV-infected people) ^b^	1,925,000	1,750,000	2,200,000
CD4 ≤350	2,600,000	2,500,000	2,800,000
CD4 ≤500^c^	4,100,000	3,950,000	4,340,000
All HIV-positive people	5,600,000	5,400,000	5,900,000
Coverage,%			
CD4 ≤200	56	65	48
CD4 ≤350 (TB or pregnant); CD4 ≤200 (all other HIV-infected people)^b^	50	56	44
CD4 ≤350	37	39	35
CD4 ≤500^c^	24	25	22
All HIV-positive people	17	18	16
Needed to initiate ART, n			
CD4 ≤200	728,434	528,434	1,028,434
CD4 ≤350 (TB or pregnant); CD4 ≤200 (all other HIV-infected people)^b^	952,444	778,444	1,228,444
CD4 ≤350	1,628,434	1,528,434	1,828,434
CD4 ≤500^c^	3,128,434	2,978,434	3,368,434
All HIV-positive people	4,628,434	4,428,434	4,928,434

ART, antiretroviral therapy; CD4, CD4 cell count (expressed in cells/μl;); NA, not applicable.

^a^Source: WHO *Towards Universal Access* report 2010 [23].

^b^We assumed 25% of those with CD4 cell counts of 200 to 350 μl to be eligible because of pregnancy or co-infection with tuberculosis.

^c^Number of people estimated to be between those eligible at 350 cells/μl or lower and those eligible under the strategy of ART for all HIV-infected people.

Table 3 gives an overview of the number of nurse-months, doctor-months, counselor-months, total FTEs, and associated salary costs required for scaling up to universal access under different eligibility criteria. Initiating treatment for all patients with CD4 cell counts of ≤350 cells/μl within one year and maintaining them on treatment for another 12 months would require a net increase of 2,200 nurses, 3,800 counselors, and 300 doctors, costing ZAR 929 million (US$ 141 million) in salaries, which is 7% of the current HIV sector budget in South Africa (Table 3). ART for all HIV-infected people will require an additional 6,000 nurses, 11,000 counselors, and 800 doctors and an additional ZAR 2.6 billion (US$ 400 million) in salaries to cover all the HHWs needed to initiate and retain ART those who are not yet on treatment, which is a 20% increase in the current total HIV sector budget of South Africa. The HHW: patient ratio for all treatment eligibility criteria is 0.2 doctors per 1,000 patients (for performing all initiations), and 1.2 nurses per 1,000 patients plus 2.1 counselors per 1,000 patients (for performing initiations and maintaining all eligible patients on treatment).

**Table 3:** Human resources needs and salary costs for initiating all those eligible for antiretroviral therapy (ART) and maintaining them on ART for one year, under different treatment strategies

	**Human resources needs**						**Costs**	
	**Nurse- months × 1,000 (95% CI)**	**Nurse FTEs × 1,000 (95% CI)**	**Counselor months × 1,000 (95% CI)**	**Counselor FTEs × 1,000 (95% CI)**	**Doctor- months × 1,000 (95% CI)**	**Doctor FTEs × 1,000 (95% CI)**	**Total salary costs, in million ZAR) (95% CI)**	**Proportion of current HIV sector budget, % (95% CI)**^**a**^
**Point estimate**^b^								
CD4 ≤200	12 (8 to 16)	1.0 (0.7 to 1.3)	20 (12 to 28)	1.7 (1.0 to 2.3)	2 (1 to 2)	0.1 (0.1 to 0.2)	12 (8 to 16)	12 (8 to 16)
CD4 ≤350 (TB or pregnant); CD4 ≤200 (all other HIV-infected people)	15 (10 to 20)	1.3 (0.9 to 1.7)	27 (16 to 37)	2.2 (1.4 to 3.1)	2 (1 to 3)	0.2 (0.1 to 0.2)	15 (10 to 20)	15 (10 to 20)
CD4 ≤350	26 (18 to 35)	2.2 (1.5 to 2.9)	45 (28 to 63)	3.8 (2.3 to 5.2)	3 (2 to 4)	0.3 (0.2 to 0.4)	26 (18 to 35)	26 (18 to 35)
CD4 ≤500	51 (34 to67)	4.2 (2.8 to 5.6)	87 (53 to 121)	7.3 (4.4 to 10.1)	6 (5 to 8)	0.5 (0.4 to 0.7)	51 (34 to67)	51 (34 to67)
All HIV-positive people	74 (50 to 99)	6.2 (4.2 to 8.2)	129 (79 to 179)	10.7 (6.6 to 14.9)	9 (7 to 12)	0.8 (0.6 to 1.0)	74 (50 to 99)	74 (50 to 99)
**High estimate**^b^								
CD4 ≤200	17 (11 to 22)	1.4 (0.9 to 1.8)	29 (17 to 40)	2.4 (1.5 to 3.3)	2 (1 to 3)	0.2 (0.1 to 0.2)	17 (11 to 22)	17 (11 to 22)
CD4 ≤350 (TB or pregnant); CD4 ≤200 (all other HIV-infected people)	20 (13 to 26)	1.6 (1.1 to 2.2)	34 (21 to 48)	2.8 (1.7 to 4.0)	3 (2 to 3)	0.2 (0.2 to 0.3)	20 (13 to 26)	20 (13 to 26)
CD4 ≤350	30 (20 to 39)	2.5 (1.7 to 3.3)	51 (31 to 71)	4.2 (2.6 to 5.9)	4 (3 to 5)	0.3 (0.2 to 0.4)	30 (20 to 39)	30 (20 to 39)
CD4 ≤500	54 (37 to 72)	4.5 (3.0 to 6.0)	94 (57 to 130)	7.8 (4.8 to 10.9)	7 (5 to 9)	0.6 (0.4 to 0.7)	54 (37 to 72)	54 (37 to 72)
All HIV-positive people	80 (54 to 106)	6.6 (4.5 to 8.8)	137 (84 to 191)	11.4 (7.0 to 15.9)	10(7 to 13)	0.8 (0.6 to 1.1)	80 (54 to 106)	80 (54 to 106)
**Low estimate**^b^								
CD4 ≤200	9 (6 to 11)	0.7 (0.5 to 1.0)	15 (9 to 20)	1.2 (0.7 to 1.7)	1 (1 to 1)	0.1 (0.1 to 0.1)	9 (6 to 11)	9 (6 to 11)
CD4 ≤350 (TB or pregnant); CD4 ≤200 (all other HIV-infected people)	13 (8 to 17)	1.1 (0.7 to 1.4)	22 (13 to 30)	1.8 (1.1 to 2.5)	2 (2 to 3)	0.1 (0.1 to 0.2)	13 (8 to 17)	13 (8 to 17)
CD4 ≤350	25 (17 to 33)	2.1 (1.4 to 2.7)	43 (26 to 59)	3.5 (2.2 to 4.9)	3 (2 to 4)	0.3 (0.2 to 0.3)	25 (17 to 33)	25 (17 to 33)
CD4 ≤500	48 (32 to 64)	4.0 (2.7 to 5.3)	83 (51 to 115)	6.9 (4.2 to 9.6)	6 (4 to 8)	0.5 (0.4 to 0.7)	48 (32 to 64)	48 (32 to 64)
All HIV-positive people	72 (48 to 95)	6.0 (4.0 to 7.9)	123 (75 to 171)	10.3 (6.2 to 14.3)	9 (6 to 12)	0.8 (0.5 to 1.0)	72 (48 to 95)	72 (48 to 95)

CD4, CD4 cell count (expressed as cells/μl); FTE, full-time equivalent; ZAR = South African rand.

^a^Current total expenditure: estimate of the total amount spent on preventing and treating HIV in South Africa in 2009 (ZAR 14 billion) [66].

^b^For the calculations of the point, high, and low estimates, we used the point, high, and low estimates of the number of people living with HIV in South Africa published in the most recent UNAIDS world AIDS report [66].

Differences in scale effects and productivity can affect estimates considerably (Table 4). Scale effects become especially important when considering broad eligibility criteria such as starting ART at CD4 cell counts of ≤500 cells/μl or for all HIV-infected people. In this case, overall costs and HHW needs can vary by 12% and 17% respectively, whereas if ART is started at CD4 cell counts of ≤350 cells/μl, economies of scale will only change the cost and HHW needs for universal access by 6%.

**Table 4:** Sensitivity analysis of human resources needs and salary costs for initiating all those eligible for antiretroviral therapy (ART) and maintaining them on ART for one year, under different treatment strategies

	**Human resources needs**						**Costs**	
	**Nurse- months × 1,000 (95% CI)**	**Nurse- FTEs × 1,000 (95% CI)**	**Counselor- months × 1,000 (95% CI)**	**Counselor FTEs × 1,000 (95% CI)**	**Doctor- months × 1,000 (95% CI)**	**Doctor FTEs × 1,000 (95% CI)**	**Total salary costs in million ZAR (95% CI)**	**Proportion of current HIV sector budget, % (95% CI)**^**a**^
**Most efficient clinic (clinic B)**								
CD4 ≤200	10 (7 to 13)	0.8 (0.6 to 1.1)	17 (10 to 24)	1.4 (0.9 to 2.0)	1 (1 to 1)	0.1 (0.1 to 0.1)	339 (223 to 456)	2 (2 to 3)
CD4 ≤350 (TB or pregnant); CD4 ≤200 (all other HIV-infected people)	13 (9 to 17)	1.1 (0.7 to 1.4)	22 (14 to 31)	1.9 (1.1 to 2.6)	1 (1 to 2)	0.1 (0.1 to 0.1)	445 (292 to 597)	3 (2 to 4)
CD4 ≤350	22 (15 to 29)	1.8 (1.2 to 2.3)	38 (23 to 53)	3.2 (1.9 to 4.4)	2 (2 to 3)	0.2 (0.1 to 0.2)	760 (499 to 1,019)	5 (4 to 7)
CD4 ≤500	42 (29 to 56)	3.5 (2.4 to 4.7)	73 (45 to 101)	6.1 (3.7 to 8.4)	4 (3 to 6)	0.4 (0.3 to 0.5)	1,459 (959 to 1,959)	10 (7 to 14)
All HIV-positive people	63 (42 to 84)	5.2 (3.5 to 7.0)	108 (66 to 150)	9.0 (5.5 to 12.5)	6 (5 to 8)	0.5 (0.4 to 0.7)	2,159 (1,419 to 2,899)	15 (10 to 21)
**Least efficient clinic (clinic C)**								
CD4 ≤200	15 (10 to 20)	1.2 (0.8 to 1.6)	28 (17 to 38)	2.3 (1.4 to 3.2)	2 (2 to 3)	0.2 (0.2 to 0.3)	559 (369 to 749)	4 (3 to 5)
CD4 ≤350 (TB or pregnant); CD4 ≤200 (all other HIV-infected people)	19 (13 to 26)	1.6 (1.1 to 2.2)	36 (22 to 50)	3.0 (1.8 to 4.2)	3 (2 to 4)	0.3 (0.2 to 0.4)	732 (483 to 981)	5 (3 to 7)
CD4 ≤350	33 (22 to 44)	2.8 (1.9 to 3.7)	62 (38 to 86)	5.1 (3.1 to 7.1)	6 (4 to 7)	0.5 (0.3 to 0.6)	1,250 (825 to 1,675)	9 (6 to 12)
CD4 ≤500	63 (43 to 85)	5.3 (3.6 to 7.1)	118 (72 to 165)	9.9 (6.0 to 13.7)	11 (8 to 14)	0.9 (0.6 to 1.1)	2,401 (1,584 to 3,218)	17 (11 to 23)
All HIV-positive people	94 (63 to 126)	7.9 (5.3 to 10.5)	175 (107 to 243)	14.6 (8.9 to 20.3)	16 (11 to 20)	1.3 (0.9 to 1.7)	3,552 (2,344 to 4,762)	25 (17 to 34)
**Increasing returns to scale**								
CD4 ≤200	11 (8 to 15)	1.0 (0.6 to 1.3)	20 (12 to 27)	1.6 (1.0 to 2.3)	1 (1 to 2)	0.1 (0.1 to 0.2)	404 (266 to 541)	3 (2 to 4)
CD4 ≤350 (TB or pregnant); CD4 ≤200 (all other HIV-infected people)	15 (10 to 20)	1.2 (0.8 to 1.6)	26 (16 to 36)	2.1 (1.3 to 3.0)	2 (1 to 3)	0.2 (0.1 to 0.2)	524 (345 to 702)	4 (2 to 5)
CD4 ≤350	25 (17 to 33)	2.1 (1.4 to 2.7)	43 (26 to 59)	3.5 (2.2 to 4.9)	3 (2 to 4)	0.3 (0.2 to 0.3)	871 (574 to 1,168)	6 (4 to 8)
CD4 ≤500	45 (30 to 59)	3.7 (2.5 to 4.9)	77 (47 to 107)	6.4 (3.9 to 8.9)	6 (4 to 7)	0.5 (0.3 to 0.6)	1,576 (1,039 to 2,113)	11 (7 to 15)
All HIV-positive people	62 (42 to 82)	5.2 (3.5 to 6.9)	107 (65 to 149)	8.9 (5.5 to 12.4)	8 (6 to 10)	0.7 (0.5 to 0.8)	2,196 (1,449 to 2,946)	16 (10 to 21)

CD4 = CD4 cell count (expressed as cells/μl); FTE, full-time equivalent; ZAR = South African rand.

The underlying number of patients needing treatment are based on the WHO-reported ART treatment coverage in South Africa [23] and UNAIDS-reported point estimate of the total number of people infected with HIV [66], see Table 2.

^a^Current total expenditure: estimate of the total amount spent on preventing and treating HIV in South Africa in 2009 (ZAR 14 billion) [66].

Regarding productivity, using the average time per patient and the average duration of work day observed in the most efficient clinic in our sample (clinic B), rather than the overall averages across all three clinics, resulted in a reduction in the additional number of nurses, counselors, and doctors required to achieve universal access by 16%, 16%, and 31% respectively, irrespective of the eligibility criteria. By contrast, using the productivity characteristics in our least efficient clinic (clinic C), the required additional number of nurses, counselors, and doctors increased by 26%, 36%, and 66%, respectively.

Alternative models of delivering HIV treatment and care can significantly affect the HHW needs and the costs of scaling up ART to universal access (Table 5). Nurse-initiated treatment, which has recently become legal in South Africa, will reduce the additional salary costs required by 7% compared with the base case (doctor-initiated treatment). For treatment initiation at CD4 cell counts of ≤350 cells/μl, this change would imply a reduction in salary costs of about ZAR 118 million (ZAR 929 million versus ZAR 811 million). For achieving universal access, the additional costs are about ZAR 336 million lower when nurses initiate treatment ( ZAR 2,639 million versus ZAR 2,304 million in salaries). However, the number of additional nurses needed will increase by 13% (from 1.2 to 1.3 per 1,000 patients). Decreasing the frequency of routine clinic visits for new initiations to one visit every 2 months can save up to 36% of salary costs, and will reduce the number of additional nurses and counselors required by 42%. Alternatively, maintaining the frequency of routine clinic visits at one visit per month, but reducing the frequency of nurse-attended visits has a similar effect on the number of additional nurses required as would reducing the overall frequency of clinic visits, but the cost savings are less substantial because the same number of counselors are needed in both cases.

**Table 5:** Effect of alternative models of antiretroviral therapy (ART) delivery on the HIV health worker-to-patient ratio and overall salary costs for universal access to HIV treatment in South Africa

**Scenario**	**Human resources needs per 1,000 patients (95% CI)**			**Overall reduction in salary costs, %**
	**Nurses**	**Counselors**	**Doctors**	
**Baseline**	1.2 (0.8 to 1.6)	2.1 (1.3 to 2.9)	0.2 (0.1 to 0.2)	NA
**Decrease visit frequency to:**				
Every 2 months	0.7 (0.5 to 0.9)	1.2 (0.7 to 1.6)	0.2 (0.1 to 0.2)	36
Every 3 months	0.5 (0.3 to 0.6)	0.8 (0.5 to 1.1)	0.2 (0.1 to 0.2)	50
Every 4 months	0.3 (0.2 to 0.5)	0.6 (0.4 to 0.8)	0.2 (0.1 to 0.2)	57
**Decrease frequency of nurses per visit to:**				
Every 2 months	0.7 (0.5 to 0.9)	2.1 (1.3 to 2.9)	2.0 (1.5 to 2.6)	24
Every 3 months	0.5 (0.3 to 0.6)	2.1 (1.3 to 2.9)	2.0 (1.5 to 2.6)	33
Every 4 months	0.3 (0.2 to 0.5)	2.1 (1.3 to 2.9)	2.0 (1.5 to 2.6)	38
**Nurse-initiated treatment**	1.3 (1.0 to 1.7)	2.1 (1.3 to 2.9)	NA	7

NA = not applicable.

## Discussion

In this study, we estimated for the first time the number of additional HHWs needed to achieve universal access to HIV treatment in South Africa, using data from a time and motion study. We found that universal access to ART at CD4 cell counts of ≤350 cells/μl will require South Africa to commit a further 2,200 nurses, 3,800 counselors, and 300 doctors to HIV treatment, at a cost of ZAR 929 million (US$ 141 million) in salaries. We found an average HHW:patient ratio of 0.2 per 1,000 patients for doctors (for performing all ART initiations), and 1.2 per 1,000 patients for nurses and 2.1 per 1,000 patients for counselors (for performing initiations of eligible people and maintaining them on treatment).

It is interesting to compare our empirical findings from this time and motion study with estimates based on other sources of information. Based on reports of the total numbers of health workers and patients in HIV treatment programs, Hirschhorn *et al.* estimated that in 2004 the number of doctors and nurses required to treat 1,000 HIV-infected people were 1–2 and 2–7, respectively, in different developing countries [29]. Based on recommendations ‘by experienced practitioners based on experience at sites in Kenya’, a 2004 WHO report estimated that 1 doctor and 2 nurses were needed to treat 1,000 HIV-infected people [30]. The lower HHW requirements estimate by our time and motion study may be due to the fact that we are now more than five years into the public-sector ART scale-up program in South Africa [31], and the productivity of HIV treatment delivery has increased because of scale effects and learning over time. Of course, it is also possible that the estimates differ because of quality-of-care differentials. The expert opinions elicited in the WHO report may have reflected a higher standard of quality of care than currently found in the real-life, public-sector HIV treatment program in rural KwaZulu-Natal, where this study took place. Similar to other treatment programs in the region, levels of non-retention and non-adherence in this program are substantial [32]; nevertheless, the program has been very effective in reducing HIV-related mortality [31] and increasing life expectancy [33] in this community, and can therefore be considered to have been successful, delivering relatively high quality of care.

Data on the current health workforce specifically devoted to HIV care in South Africa are not available, but WHO estimates show that in 2004 there were around 35,000 doctors and 180,000 nurses in total in the country [34]; consequently, the HW:population ratio in South Africa exceeded the threshold of 2.3 doctors and nurses per 1,000 population that has been proposed by WHO as a critical minimum [35,36]. Although our study determined the additional numbers of health workers required for future ART scale-up under different scenarios, it did not enable us to evaluate this number directly in relation to the supply of health workers in order to establish whether there is a shortage in health workers in South Africa, either nationwide or in regions. The number of doctors required to provide universal access to ART is currently one of the most important capacity constraints in SSA, because only a few thousand doctors graduate from medical schools on the entire subcontinent each year [37,38], and the rates of doctor migration to countries outside the region remains high [11]. However, scaling up treatment to universal access for initiation at CD4 cell counts of ≤350 cells/μl will require only about 300 additional doctors committed to performing ART initiations, and may thus be feasible without major changes to national health worker production and retention. However, recruiting the required additional 2,200 nurses fully devoted to HIV care may prove to be a greater challenge, given that the total of all professional nurses who graduated from nursing schools in South Africa in 2011 was only about 5,600 [39].

It is therefore vital to increase efforts to expand the health worker pool for HIV in South Africa by increasing training and retention, considering reinstatement of retired health workers, or increasing HHW productivity, in particular to achieve universal coverage at more relaxed eligibility criteria [37]. Currently, public-sector HHWs are paid a salary on a monthly basis and, additionally, receive contributions to health and retirement pension insurance, as well as a rural allowance for service in under-served areas. Alternative models of contracting and incentivizing HHWs, such as performance-based payment, might improve productivity. However, such new models might also lead to inefficiencies (for example, transaction costs for monitoring performance), and unintended behavioral consequences (for example, decreased quantity and quality of care for services not included in the performance-based payment scheme) [40]. It is possible that transferring public-sector patients on ART to the private sector for routine follow-up and monitoring, as has been done effectively in Botswana and Mexico [41,42], might increase the pool of available HHWs. At the same time, of course, this strategy might increase the human resources costs per individual on ART, because health worker salaries in the private sector in South Africa are higher than those in the public sector. Health worker interventions, such as shifting tasks from more to less skilled health workers [43] and integration of ART delivery into the general primary care services [44] should also be considered as means to free up human resources for HIV treatment. Integration might improve productivity, if it either increases capacity utilization (for example, reducing idle time) or leads to economies of scope, as different health services are combined. Redistribution of HHWs from overstaffed to understaffed clinics could further improve HHW productivity by reducing idle time. Finally, a number of interventions could increase the supply of health workers in South Africa, nationally or in regions, including interventions to decrease health worker migration out of the country and to increase health worker production [45,46]. One recent example is the 2012 agreement between Cuba and South Africa, which is intended to ensure continued placement of Cuban doctors in South African medical schools and increased training of South African nationals in Cuban medical schools [47].

There are several options for improving HHW productivity in PHC HIV clinics. We found that the average duration of a work day varied widely between clinics. The number of HHWs required for the treatment of 1,000 ART patients could be reduced by about one-third if all clinics achieved the productivity of the most efficient clinic. In addition, the average duration of a work day in our data was 6.3 hours. If we assume an HHW work day to last 8 hours, we find that the total number of nurses, counselors, and doctors required would be reduced by 23%, 24%, and 37% compared with the baseline in clinics A, B, and C, respectively. Another way to improve productivity might be to reduce opening hours in selected clinics. We found that in the least busy (and most rural) clinic in our sample, only 20% of HHW time on one particular day was spent on direct patient contact and 36% was spent on breaks and idle time, because only a few patients visited the clinic on that day. Limiting the opening hours of selected PHC HIV clinics might reduce wastage due to idle time. At the same time, however, such a strategy carries the potential danger of reducing access for particular populations, such as employed people or people living in rural areas. Finally, productivity gains might be achieved by cutting the time spent on breaks and idle time between patients. However, we found that on average, only 9% of the work day was spent in this category, which translates to 43 minutes of break time in a work day of 8 hours, which is very short considering the demands of the job and the need for health staff to eat and take care of themselves during the work day, thus there is little room for considerable productivity gains through this approach.

Universal access for all HIV-infected people (’treatment as prevention’) would require an additional 800 doctors, 6,000 nurses, and 11,000 counselors fully dedicated to HIV treatment. Salary costs to cover all HHWs under such a strategy would be about ZAR 2.6 billion (US$ 394 million) per year, which is an increase of 20% compared to the current total HIV sector budget in South Africa. Of course, salaries are only one part of the running costs of added HHWs and expansion of HIV treatment. Other expenditures necessary for HIV treatment include running costs for drugs, medical supplies, water and heating, and investment costs for equipment, facilities, and continuing HHW education [48,49]. In addition, the need for support staff not involved in direct patient contact, such as laboratory technicians, supply-chain workers, general management staff, and trainers, will also grow with increasing treatment coverage. At present, a strategy of providing ART for all HIV-infected people seems unrealistic for South Africa without large additional financial commitments and substantial increases in HHW training capacity. However, the future effects of a `treatment as prevention’ strategy through reduced transmission has been suggested to be considerable [15,50,51], possibly outweighing the initial investments. Furthermore, the workload of initiating patients with advanced disease is higher than for patients with relatively high CD4 cell counts, resulting in a lower HHW:patient ratio. Further research is needed into the epidemiological and clinical benefits of a ‘treatment as prevention’ strategy, for example, through randomized controlled trials in general populations in SSA [52].

Alternative models of ART delivery through task shifting or simplifications might provide a solution for the shortage of HHWs. Preliminary studies have shown that the quality of nurse-initiated treatment is comparable with that of doctor-initiated treatment [53], and this strategy has recently been implemented in South Africa [13]. Our results suggest that this strategy could reduce overall salary costs by 7%. Reducing the required frequency of routine clinic visits could also be a useful tool to decrease the required number of HHWs required to maintain patients on ART. Currently, patients are required to visit the clinic every month until treatment has stabilized, and once every 2 months thereafter. Up to 50 to 60% of total salary costs might be saved if patients visited a clinic only once every 3 or 4 months, or if the number of nurse-attended visits were decreased. Although these figures suggest substantial potential for resource savings for universal HIV treatment coverage, it is currently not understood how the implementation of these alternative treatment delivery models will affect the quality of care. Levels of non-adherence and non-retention are already worryingly high in South Africa’s expanding ART program [54], and these problems might worsen if routine-visit frequencies were decreased.

Our study has several limitations. First, the time and motion data used in this study were from three different PHC HIV clinics in a rural area, and covered only 13 days of observation. However, the time spent per patient did not differ significantly between the clinics. Although the duration of the work day did differ significantly between two of the clinics, this could have been caused by the location of the clinics (rural versus more urban), which is associated with patient load, rather than with differences in work routine or the full potential productivity of the HHW. In the clinic with the shortest effective work day, an average of 14% of the time was spent on breaks and idle time between patients, whereas in the other clinics this percentage was only 5% and 7%.

Second, we extrapolated our data from this specific rural setting to South Africa as a whole. Similar studies in other parts of South Africa are needed to confirm whether this extrapolation is justified.

Third, HHWs were aware that they were being observed, which might have induced them to increase their productivity during observed visits compared with unobserved visits. However, such a Hawthorne effect [55] is likely to be limited, for a number of reasons. The observer was not involved in the HIV treatment program and was not known to the HHW. Moreover, the observer completely abstained from providing performance feedback to the observed HHW, even if asked for such feedback, so as to eliminate this underlying cause of the Hawthorne effect [56]. Finally, we did not detect substantial changes in time allocation patterns within individual clinics between the different observation days, suggesting that observer bias, which is likely to wane (owing to increasing HHW tolerance to being observed) or grow (owing to feedback and learning) over time, may not have played an important role in this study.

Fourth, the observed activities might not be all-encompassing, as some infrequent activities such as continued training and attending workshops or conferences were not observed. However, these activities will take up only a limited proportion of the total number of work hours over a year [39] and any bias resulting from excluding these activities will thus be limited.

Finally, there are several assumptions in our analyses that might hold precisely true. We assumed that the measured doctor time was only for initiations, while the counselor and nurse time was for both initiation and follow-up. This simplification could have biased our estimates, especially for doctor time, as doctors also perform tasks other than initiations, such as completing disability grant forms or looking after more complex cases (for example, suspected treatment failure, side-effects, or adverse events).

Task time allocation and HHW productivity is likely to depend on the model of HIV treatment delivery. The current delivery model may change, because South Africa has embarked on a major reform of PHC, with the goal of providing universal access to a comprehensive package of healthcare services in the public sector through a national health insurance scheme [57]. Several initiatives included in the reform may improve the availability of HHWs. So-called district specialist teams will be recruited and deployed in all of the 52 districts in the country to improve the standard of care delivered in community-based healthcare facilities [39]. These teams will consist of four medical specialists and three advanced professional nurses. In addition, each PHC ward will have at least one PHC outreach team consisting of a professional nurse, environmental health and health promotion practitioners, and four to five community health workers. The role of the outreach team will include health promotion and prevention campaigns, early detection and interventions for selected health problems, and support for treatment retention and adherence [39]. These initiatives, which are currently tested in pilot studies, could help to further devolve HIV treatment to communities and homes, freeing up HHWs in healthcare facilities.

We provide a point estimate for the HHWs required for one year of treatment at the present time. In the longer run, the current HHW and ART coverage levels may themselves affect HHW requirements, for a number of reasons [38]. On the one hand, because ART is effective in reducing mortality, the more patients receive ART in the current period, the more patients will require treatment in future periods, assuming that HIV incidence remains unchanged [58,59]. On the other hand, ART can also effectively prevent HIV transmission [15,50,51,60-62], which would lead to a reduction in the number of future cases, and thus a reduction in the number of HHWs required in future periods. Dynamic models are needed in future studies to examine the effects of the mortality-reduction and transmission-prevention effects of ART on long-term HHW requirements. Those studies should also take into account that ART may change the type of patient needing ART. For example, ART may shift the age composition of patients on ART towards older ages [63,64], increasing the average morbidity among ART patients and the average health worker time required for providing appropriate treatment for each patient. Moreover, the case mix of patients on ART might change over time due to increasing rates of ART failure and long-term ART toxicities. These changes could increase the average health worker time required per patient because they necessitate time-consuming counseling, ART switches, and complex treatments for the side-effects of ART [65].

## Conclusion

We provide policy-relevant estimates of the number of HHWs needed and associated salary costs for scaling up HIV treatment to achieve universal access under different treatment eligibility criteria and delivery models in South Africa. We show that, in terms of HHWs required for scaling up ART to universal access at CD4 cell count of ≤350 cells/μl seems achievable in the present context, whereas universal coverage for all HIV-infected people is likely to be extremely difficult unless substantial additional human and financial resources can be mobilized for ART delivery. Further research is needed to determine how different treatment strategies affect the movement of patients in and out of the system through reduced transmission and increased survival, and how the quality and productivity of HIV treatment is affected by different ART delivery models.

## Competing interests

The authors declare that they have no competing interests.

## Authors’ contributions

ML, TB, and RB were involved in conceptualization of the study; TB, RB, and RL implemented and oversaw the study; KM collected the data; RL supervised the data collection; TB informed the analysis plan; JH performed all analyses; and JH wrote the initial draft of the manuscript. All authors contributed to drafting the final manuscript. All authors read and approved the final version of the manuscript.
